# Knowledge, Attitudes and Referral Patterns of Lynch Syndrome: A Survey of Clinicians in Australia

**DOI:** 10.3390/jpm4020218

**Published:** 2014-05-12

**Authors:** Yen Y. Tan, Amanda B. Spurdle, Andreas Obermair

**Affiliations:** 1 School of Medicine, The University of Queensland, 288 Herston Road, Herston, QLD 4006, Australia; E-Mail: obermair@powerup.com.au; 2 Molecular Cancer Epidemiology Laboratory, Genetics and Computational Biology Division, QIMR Berghofer Medical Research Institute, 300 Herston Road, Herston, QLD 4006, Australia; 3 Queensland Centre for Gynaecological Cancer Research, Level 6 Ned Hanlon Building, Royal Brisbane and Women’s Hospital, Butterfield Street, Herston, QLD 4029, Australia; E-Mail: amanda.spurdle@qimrberghofer.edu.au

**Keywords:** Lynch syndrome, referral, risk assessment, genetic services, knowledge, attitudes and practice

## Abstract

This study assessed Australian clinicians’ knowledge, attitudes and referral patterns of patients with suspected Lynch syndrome for genetic services. A total of 144 oncologists, surgeons, gynaecologists, general practitioners and gastroenterologists from the Australian Medical Association and Clinical Oncology Society responded to a web-based survey. Most respondents demonstrated suboptimal knowledge of Lynch syndrome. Male general practitioners who have been practicing for ≥10 years were less likely to offer genetic referral than specialists, and many clinicians did not recognize that immunohistochemistry testing is not a germline test. Half of all general practitioners did not actually refer patients in the past 12 months, and 30% of them did not feel that their role is to identify patients for genetic referral. The majority of clinicians considered everyone to be responsible for making the initial referral to genetic services, but a small preference was given to oncologists (15%) and general practitioners (13%). Patient information brochures, continuing genetic education programs and referral guidelines were favoured as support for practice. Targeted education interventions should be considered to improve referral. An online family history assessment tool with built-in decision support would be helpful in triaging high-risk individuals for pathology analysis and/or genetic assessment in general practice.

## 1. Introduction

Lynch syndrome, also known as hereditary nonpolyposis colorectal cancer (HNPCC), is an inherited cancer syndrome caused by defect in one of the mismatch repair (MMR) genes—*MLH1*, *MSH2*, *MSH6* and *PMS2.* It accounts for about 5% of all colorectal and endometrial cancers diagnosed [1,2]. MMR mutation carriers have high risks of early onset colorectal (25%–70%) or endometrial cancers (30%–70%), and have increased risks of other cancers such as ovarian, gastric, small bowel, pancreatic, urothelial, brain and skin neoplasms [3,4]. Further, women carrying MMR gene mutations who are diagnosed with endometrial cancer have increased risks of second primary colon cancer (about 40-fold) or other extracolonic cancers (up to 28-fold) compared with that for the general population [5]. Nevertheless, at-risk individuals are dependent on their clinicians for diagnosis and surveillance. Though clinicians cannot be expected to have detailed knowledge about the causative genes of Lynch syndrome, it remains their responsibility to recognize the clinical phenotype and family history characteristics of Lynch syndrome, and make a referral to a clinical genetics service or a familial cancer centre if deemed necessary. However, local and international studies have reported that only a small proportion of individuals suspected to have Lynch syndrome were identified and referred to a clinical genetics service/family cancer clinic for further genetic consultation and possible genetic testing [6,7,8,9,10].

Numerous barriers to referral for genetic services (*i.e.*, genetic consultation and/or testing) have been reported in the literature, including lack of knowledge regarding Lynch syndrome and who should be referred, lack of family history information or referral guidelines, or lack of awareness of clinical genetic services [11,12]. Despite the number of studies on Lynch syndrome, only few included Lynch-associated extracolonic malignancies [13,14]. This contributes to the concern that, although there is increasing awareness of Lynch syndrome and colorectal cancer, endometrial and other extracolonic cancers are under-recognized by practicing clinicians. Domanska and colleagues investigated knowledge about key features of Lynch syndrome in 102 clinicians in Southern Sweden reported that not only a majority of clinicians underestimated the risk of endometrial cancer (77%) but also that of colorectal cancer (56%) [14]. In a more recent survey about the knowledge of regarding the genetics and recommended screening for carriers of Lynch syndrome mutations, 201 medical students at an American medical school demonstrated lack of awareness of recommendations for endometrial cancer screening for high risk individuals [15]. As it was unclear whether clinicians in Australia face similar challenges as their peers abroad, a qualitative study was conducted to assess barriers and motivators of genetics referral among 28 clinicians who are likely to diagnose, treat and assess Lynch families in Queensland, the second-largest and third-most populous state in Australia [16]. While the authors reported that a majority of clinicians were positive about referring patients to a clinical genetics service, they also found a lack of knowledge and support needed to make an appropriate referral.

In order to facilitate diagnosis of Lynch syndrome, various guidelines such as the Amsterdam II, revised Bethesda or the Society of Gynecologic Oncologists have been developed [17,18,19]. Current Australian practice guidelines recommend that young individuals with colorectal cancer, and with a moderate-to-high risk family history of cancer are referred to a clinical genetics service/family cancer clinic, and offered genetic risk assessment and counselling with or without genetic testing *i.e.*, DNA molecular analysis [20]. While the referral of patients to a clinical genetics service/family cancer clinic can be from a general practitioner (GP) or a specialist [21], only specialists apart from geneticist/genetic counsellor can initiate or order tumour molecular analysis *i.e.*, immunohistochemistry (IHC) or microsatellite instability (MSI) testing directly from a pathology service. Routine IHC for MLH1, MSH2, MSH6 and PMS2 proteins has been recommended and supported by the Australian College of Pathologist for all patients diagnosed with colorectal cancer younger than 50 years of age [22], although the actual patterns of care around uptake of this recommendation in Australia is unknown. Further, to our knowledge, such testing has yet to be formally established in Australia and there are currently no standard criteria for referral of individuals with endometrial cancer who are at risk of Lynch syndrome to a clinical genetics service. Genetic testing, on the other hand, is done through clinical genetics services or family cancer clinics, and is free if a patient presents with a high clinical suspicion of an underlying genetic aetiology.

As early identification of mutation carriers allows for more intensive colonoscopic surveillance and consideration of risk-reducing surgeries [23,24,25,26,27], it is important that patients diagnosed with colorectal cancer or endometrial cancer are identified and appropriately triaged for clinical evaluation. We therefore distributed a questionnaire to: (1) investigate clinicians’ knowledge of Lynch-associated cancer risks and tumour molecular analysis; (2) assess referral practices among clinicians who are likely to diagnose, treat and survey Lynch families; (3) quantify motivators and barriers to genetics referral for Lynch syndrome in Australia; and (4) explore physician referral preferences (*i.e.*, preferred timing for referral and who they think might be the most appropriate professional to make the initial genetics referral), perceptions of their role and their desired support for the provision of genetic services.

## 2. Experimental

### 2.1. Participants and Procedures

We targeted our sample to clinicians who are likely to diagnose, treat and survey Lynch families. Participants therefore included GPs and specialist groups (*i.e.*, gynaecologists, gastroenterologists, medical oncologists, radiation oncologists, gynaecology oncologists, general and colorectal surgeons), who were identified through membership list of the Australian Medical Association. We purchased the list from AMPCo Data direct, a subsidiary of the Association who owns the list of practicing doctors (N = 11,624), who then broadcasted our invitation to their members. An invitation was circulated by email to a randomly stratified sample of 1674 participants. Sample size was calculated to provide 90% power, with a two-sided significant level of α = 0.06. The email described the study and confidentiality, and included a link to the online survey and participant information sheet. Participants were asked to read the information brochure prior to commencing the survey. A reminder email was sent a week apart from the initial broadcast. Responders were allowed to save their responses and return to complete the survey at a later time. Due to the small sample size of oncologists from the Australian Medical Association database, oncologists were also identified from the membership of Clinical Oncological Society of Australia membership directory. An invitation email was sent to gynaecological oncologists, medical oncologists and radiation oncologists by the Society, and was followed-up with a reminder email sent a week after the initial broadcast. Only one reminder email was sent to all invited study participants. Participation was voluntary and no financial incentives were given. Survey response implied consent to participate, and all responses were anonymous. The study was approved by The University of Queensland School of Medicine and Royal Brisbane & Women’s Hospital research ethics committees.

### 2.2. Instrumentation

A web-based survey was developed specifically for this study based on a review of relevant literature [13,28,29,30,31,32], results from our previous qualitative study [16], and using the advice of a panel of experts with expertise in gynaecology oncology, clinical genetics, psycho-oncology, and genetic counselling. The survey items were pilot-tested with a convenience sample of 10 health professionals (*i.e.*, oncologists, geneticists, clinical and social researchers) to assess relevance and face validity of survey items. Changes to the survey were made accordingly using the Delphi method [33] before administration.

The final version of the survey was created using LimeSurvey and consisted of 19 items (see Appendix 1). Items included participant demographics, referral practices, barriers and motivators for genetics referral, physician referral preferences and perceptions of their role and their desired support for the provision of genetic services. In order to evaluate clinicians’ knowledge of Lynch syndrome, the authors adapted a pedigree from a previous study that addressed risk assessment abilities and referral patterns to fit the high-risk profile for Lynch syndrome [28]. At the end of the survey, participants were asked to provide additional comments and their email address if they wish to receive a copy of the research summary report. The survey was open for three months from mid-March to end of May 2013.

### 2.3. Data Analyses

All survey data were collected using LimeSurvey, and data were coded and analysed using SPSS Version 20 (IBM^®^ SPSS^®^ Statistics, Chicago, IL, USA). Descriptive statistics included frequencies and proportions were calculated. *x*^2^ or Fisher’s exact test was used to investigate associations between categorical variables. Throughout the analysis, respondents who selected unsure were designated as missing data. All p-values were two-sided, with a statistical significance level set at *p* < 0.05.

## 3. Results

Of 582 email recipients who “opened” the email, 144 (24.7%) responded to the web survey fully (138/144, 95.8%) or partially (more than 50% of questions answered, 6/144, 4.2%). Demographics of these clinicians are summarized in Table 1. Overall, the study sample consisted of 59 oncologists (40%), 27 surgeons (19%), 24 gynaecologists (17%), 18 GPs (13%) and 11 gastroenterologists (8%); the remaining 5 were designated as other specialties (4%). Most clinicians were <50 years of age (*p* = 0.005), and were in practice for at least 10 years (*p* = 0.002). There were no statistically significant differences between gender or state of residence with provider groups.

**Table 1:** Sociodemographics of participating health care provider groups ^a^.

	Total	GP	GYN	GE	ONC	SURG	Others ^b^
	N (%) ^c^	N (%) ^c^	N (%) ^c^	N (%) ^c^	N (%) ^c^	N (%) ^c^	N (%) ^c^
**Total**	144 (100)	18 (100)	24 (100)	11 (100)	59 (100)	27 (100)	5 (100)
**Age (years)**							
<50	**84 (58)**	10 (56)	11 (46)	6 (55)	37 (63)	17 (63)	3 (60)
≥50	**60 (42)**	8 (44)	13 (54)	5 (46)	22 (37)	10 (37)	2 (40)
**Gender**							
Female	67 (47)	8 (44)	8 (33)	**0**	**37 (63)**	9 (33)	**5 (100)**
Male	77 (53)	10 (56)	16 (67)	**11 (100)**	**22 (37)**	18 (67)	**0**
**Years of Practice in Specialty**							
<10	59 (41)	5 (28)	7 (29)	4 (36)	27 (46)	12 (44)	4 (80)
≥10	85 (59)	13 (72)	17 (71)	7 (64)	32 (54)	15 (56)	1 (20)
**State ^d^**							
NSW/ACT	44 (31)	6 (33)	**9 (38)**	**0**	18 (31)	9 (33)	3 (60)
VIC/TAS	44 (31)	2 (11)	**5 (21)**	**4 (36)**	20 (34)	11 (41)	2 (40)
QLD	29 (20)	5 (28)	**11 (46)**	**2 (18)**	9 (15)	3 (11)	0
SA	11 (8)	2 (11)	**0**	**3 (27)**	6 (10)	0	0
WA	16 (11)	3 (17)	**1 (4)**	**2 (18)**	6 (10)	4 (15)	0

**Abbreviations: GPs** general practitioners; **GYNs** gynaecologists; **GEs** gastroenterologists; **ONCs** oncologists; **SURGs** surgeons; **NSW** New South Wales; **ACT** Australian Capital Territory; **VIC** Victoria; **TAS** Tasmania; **QLD** Queensland; SA South Australia; **WA** Western Australia ^a^ Bolded estimates indicate statistically significant difference between two groups within each practice category; ^b^ Other medical specialties include cancer care coordinator (*n* = 1), genetic counsellor (*n* = 1), psycho-oncologists (*n* = 2), social worker (*n* = 1); ^c^ The % reflects the percentage responding within each practice category; ^d^ ACT and TAS were consolidated with NSW and VIC, respectively, due to low participation rate and similarity in genetic testing protocols.

### 3.1. Referral to Genetics Services and Ordering Diagnostic Testing for Lynch Syndrome

Table 2 displays clinician likelihood to refer patients to genetic services and ordering diagnostic testing for Lynch syndrome in the past 12 months, by the provider group. Overall, there were no significant differences between specialist groups and likelihood to refer to genetic services. However, GPs, particularly male practitioners who have been in practice for more than 10 years, were less likely to refer patients to genetic services than others (*x*^2^ = 16; *p* = 0.001; 25% *vs.* 87%). GPs and gynaecologists were significantly less likely to order IHC testing (*p* = 0.002 and *p* = 0.003, respectively), whereas oncologists and surgeons were more likely to order such testing (*p* = 0.03 and *p* = 0.004, respectively). GPs and gynaecologists were also less likely to order MSI testing (*p* = 0.003 and *p* = 0.004, respectively), but surgeons were more likely than the others to order MSI testing (*p* = 0.005). Oncologists were more likely than other provider groups to order DNA germline testing (*p* = 0.007), whereas gynaecologists were less likely to do so. There were no statistically significant differences between age, gender, state of residence or years in practice and referral to genetic services.

**Table 2:** Likelihood that clinicians reported referring patients to clinical genetic services or ordering tumour analysis in the past 12 months, by provider group ^a,b^.

	Total (N = 144)	GP (N = 18)	GYN (N = 24)	GE (N = 11)	ONC (N = 59)	SURG (N = 27)	Others ^c^ (N = 5)
	N(%; 95%CI) ^d^	N(%;95%CI) ^d^ *p*-value	N(%;95%CI) ^d^ *p*-value	N(%;95%CI) ^d^ *p*-value	N(%;95%CI) ^d^ *p*-value	N(%;95%CI)^d^ *p*-value	N(%;95%CI) ^d^ *p*-value
Referred patients to genetic services	112 (78; 70–84)	**9 (50; 29–71) 0.003**	19 (79; 60–91) 0.969	10 (91; 62–98) 0.458	50 (85; 73–92) 0.095	22 (81; 63–92) 0.799	2 (50) 0.196
Ordered tumour IHC testing	77 (53; 45–61)	**4 (22; 9–45) 0.002**	**6 (25; 12–45) 0.003**	8 (73; 43–90) 0.524	**39 (66; 53–77) 0.030**	**20 (74; 55–87) 0.004**	0 -
Ordered tumour MSI testing	55 (38; 31–46)	**1 (6; 1–26) 0.002**	**3 (13; 4–31) 0.004**	6 (55; 28–79) 0.527	29 (49; 37–62) 0.075	**16 (59; 41–75) 0.005**	0 -
Ordered DNA mutation testing	67 (47; 39–55)	6 (33; 16–56) 0.125	**7 (29; 15–49) 0.034**	3 (27; 10–57) 0.308	**36 (61; 48–72) 0.007**	15 (56; 37–72) 0.249	0 -

**Abbreviations**: **GP** general practitioners; **GYN** gynaecologists; **GE** gastroenterologists; **ONC** oncologists; **SURG** surgeons; **CI** confidence interval; **IHC** immunohistochemistry; **MSI** microsatellite instability. ^a^ Category totals may be less than the total number of respondents due to missing values; ^b^ Bolded estimates indicate significant findings; ^c^ Other medical specialties include cancer care coordinator (*n* = 1), genetic counsellor (*n* = 1), psycho-oncologists (*n* = 2), social worker (*n* = 1); ^d^ The % reflects the percentage responding within each practice category.

### 3.2. Risk and Surveillance Strategies among Clinicians

When participants were presented with a fictitious high-risk Lynch-specific clinical scenario (Appendix 1), only 13/144 (9%) of all respondents were able to answer all Lynch cancer risk-associated questions correctly, *i.e.*, no elevated risk for breast or thyroid cancer, somewhat higher risk for ovarian and gastric cancer, and much higher risk for colorectal and endometrial cancer as compared to the general population. Of all respondents, 65% could correctly identify colorectal cancer risk, followed by 63% for thyroid cancer, 60% for endometrial cancer, 45% for gastric cancer and 42% for ovarian cancer. About a third of all respondents considered the fictitious high-risk asymptomatic individual to be at lower risk of developing colorectal and endometrial cancer when compared to the general population, but half of all respondents considered the individual to have a much higher risk for developing ovarian cancer, and another 15% considered the individual to have a much higher risk for developing gastric cancer. A notable 44% considered the individual to have high risk for breast cancer. There were no statistically significant differences in specialist groups, age, gender, state of residence or years in practice with providing the correct answers. Nevertheless, GPs were less likely than other specialists to recognize high risk for colorectal cancer (*x*^2^ = 9; *p* = 0.003; 33% *vs.* 69%) and endometrial cancer (*x*^2^ = 6; *p* = 0.015; 33% *vs.* 64%).

When asked if they consider IHC or MSI testing a germline test, 9% of all respondents thought IHC was a germline test, while another 23% considered MSI a germline test. Forty-nine percent and 35% of all respondents did not consider IHC or MSI a germline test, respectively. Forty-two percent of all respondents were uncertain about both tests. GPs (89%) and gynaecologists (75%) were less likely to answer the question on IHC correctly (*p* < 0.05 for both groups), while oncologists (68%) were more likely to answer the question correctly (*p* < 0.001) and refer patients for genetic services (*p* = 0.008).

When a high risk individual was affected with endometrial cancer, respondents indicated they would: assess family history (96%); refer to a geneticist (94%); discuss Lynch syndrome cancers with the patient (82%); offer cancer surveillance (77%); order IHC or MSI tumour testing (58%); discuss risk-reducing surgeries (53%); order germline testing (42%); and refer to non-genetics specialists (38%); and no further action (1%). For those who offered cancer surveillance and discuss risk-reducing surgeries, they recommended colonoscopy (87%), gastroscopy (62%), serum CA125 (48%), hysterectomy and bilateral salpingo-oophorectomy (41%), breast ultrasound (36%), subtotal or segmental colectomy (8%), pelvic ultrasound (7%) and mastectomy (4%).

### 3.3. Prevalence of Motivators and Barriers to Referral

As shown in Table 3, a number of motivators and barriers for referral to genetic services were examined. Patient disinterest was considered the biggest barrier to referral. Clinicians that have never referred patients for genetic services were more likely to denote the following barriers to referral: unfamiliarity with hereditary cancer syndromes (*p =* 0.04); no knowledge of how to make referral (*p* ≤ 0.001); no access to a genetic health service (*p* = 0.001); no recommendation and guidelines for referral (*p* = 0.016).

**Table 3:** Motivators and barriers of referral of patients suspected with Lynch syndrome for genetic services *.

	Have not Referred for Genetic Services (Total = 30) N (%) ^a^		Referred for Genetic Services (Total = 112) N (%) ^a^		*p-value *^b^
**Motivators**					
To provide genetic counselling for the patient	20	(66)	103	(92)	**<0.001**
Patient interest or request	20	(66)	92	(82)	0.065
To provided appropriate screening and/or management for the patient’s family	15	(50)	94	(84)	**<0.001**
To provide appropriate cancer risk assessments for the patient	21	(70)	98	(88)	0.544
To provide genetic testing for germline mutations	14	(47)	83	(74)	**0.004**
Reassurance for the patient and family	16	(53)	73	(65)	0.234
Ethical and legal responsibility	14	(47)	70	(63)	0.117
To provide appropriate screening and management for the patient	20	(66)	81	(72)	0.544
Others ^c^	0		5	(4)	-
**Barriers**					
Patient was not interested when referral was offered	17	(57)	62	(55)	0.898
Patient may be at risk for insurance discrimination	5	(17)	19	(17)	0.969
Recommendations and guidelines were not available to select patients for referral	8	(27)	11	(10)	**0.016**
Patient is unlikely to benefit from genetic counselling/testing	1	(3)	16	(14)	0.123
I do not feel familiar with hereditary cancer syndromes	5	(17)	6	(5)	**0.040**
Long waiting time for appointment at genetics clinic	2	(7)	7	(6)	0.934
I do not know how to make a referral to the local genetic health service	7	(23)	1	(1)	**<0.001**
I do not have access to genetic health service	6	(20)	2	(2)	**0.001**
I do not feel it is my responsibility	2	(7)	1	(1)	0.113
Others ^d^	0		4	(4)	-

* For referral to genetic services, total respondents were 142 in total. ^a^ The % reflects the percent responding within each category; ^b^ Bolded estimates indicate significant findings between clinicians who have referred (*n* = 112) and did not refer (*n* = 30) patients for genetic services; ^c^ Qualitative answers were provided, which include antenatal diagnosis, significant family history, significant tumour testing results, to provide the necessary routine surveillance and advice regarding screening and risk-reducing options for patients; ^d^ Qualitative answers were provided, which include lack of resources and no prophylactic treatment or screening available for patients.

### 3.4. Referral preferences, Perceived Role and Desired Support for the Delivery of Genetic Services among Clinicians

**Figure 1:**
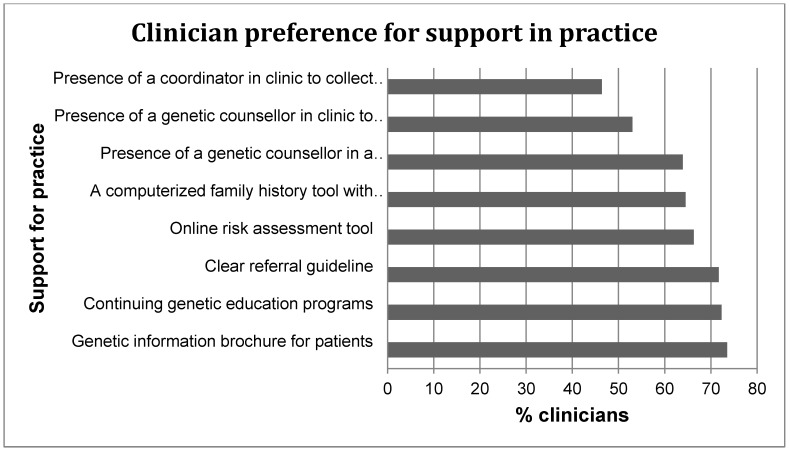
Desired support for the delivery of genetic services among health care providers.

There was no statistically significant difference between provider groups and preferred timing for referral. Clinicians indicated referral should be warranted at any time (51%) and when family history is collected (27%). Although a majority of clinicians felt that everyone is responsible for referring patients to genetic services, a proportion of clinicians believe that oncologists (15%) and GPs (13%) would be best suited for the role (*p* = 0.031). Oncologists were preferred as they would most likely recognize at-risk individuals, whereas GPs were preferred because they are the first point of contact for patients, who have the best knowledge about patients’ family history and are the ones responsible for coordinating care. However, compared to the specialist groups, some GPs did not feel that their role is to identify patients for referral to genetic services (*x*^2^ = 7; *p* = 0.011; 30% *vs.* 10%) or to order IHC or MSI testing (*x*^2^ = 11; *p* = 0.001; 20% *vs.* 2%). Table 4 further shows clinicians’ perceived roles and referral for genetic services. Clinicians who have referred for genetic services considered themselves to be responsible for (1) identifying and referring individuals to clinical genetics services; (2) interpreting germline DNA mutation testing results; (3) ordering IHC or MSI tests; (4) discussing cancer surveillance and prophylaxis with patients; and (5) providing regular clinical exams and care to patients with hereditary cancer syndromes. The support desired for delivering genetic services is summarized in Figure 1.

**Table 4:** Clinicians’ perceived roles and referral of patients suspected with Lynch syndrome genetic services *.

Perceived Roles	Have not referred for genetic services (Total = 30) N (%) ^a^	Referred for genetic services (Total = 112) N (%) ^a^	*p*-value ^b^
Providing emotional support after genetic testing	21 (70)	64 (57)	0.202
Identifying patients for referral to genetic services	19 (63)	104 (93)	**<0.001**
Interpreting germline DNA-based genetic test results	0	22 (20)	**0.004**
Collecting a three-generation family history information	11 (37)	54 (48)	0.260
Ordering pre-genetic testing of tumour tissue (e.g., MSI or IHC)	5 (17)	53 (47)	**0.002**
Counselling patients about their cancer risks after genetic testing	8 (27)	38 (34)	0.450
Counselling patients about their cancer risks before genetic testing	7 (23)	42 (38)	0.147
Calculating relative risk of cancer associated with family cancer history	4 (13)	25 (22)	0.321
Discussing the need for cancer surveillance or prophylaxis with patients when required	17 (57)	90 (80)	**0.011**
Providing regular clinical examination and care to patients with hereditary cancer syndromes	15 (50)	82 (73)	**0.015**
Other	3 (10) ^c^	5 (5)	-

* For referral to genetic services, total respondents were 142 in total. ^a^ The % reflects the percentage responding within each category; ^b^ Bolded estimates indicate significant findings between clinicians who have referred (*n* = 112) and did not refer (*n* = 30) patients for genetic services; ^c^ Qualitative answers were provided, which include liaison with genetic services, psychotherapy and specialty specific to one particular general surgeon; ^d^ Qualitative answers were provided, which include advice on sequence of treatment modalities, antenatal advice and diagnosis, counselling provided in addition to that provided by genetic service, clear documentation to clinicians, ensuring patient informs family of risk, and referring to appropriate services.

## 4. Discussion

In 2009, a total of 2105 patients were diagnosed with endometrial cancer in Australia [34]. As the prevalence of Lynch-associated endometrial cancer among mutation-positive women is comparable to that reported internationally [35], it would be expected that about 100 cases diagnosed annually would be due to Lynch syndrome. Establishing a pathogenic mutation carrier in a family is important since it enables predictive testing to be offered to family members, and allow decisions to be made about screening, risk-reducing surgery and chemoprevention if found to be MMR mutation positive [23,24,25,26]. While genetic testing is typically offered free of charge and always in the context of genetic counselling at an accredited clinic in Australia [36], access to publically funded genetic services requires a referral from either a GP or a medical specialist [37]. It is therefore essential that clinicians in general recognize the classical family history indicators for Lynch syndrome when individuals present in everyday practice. To our knowledge, this is the first study to formally examine the knowledge, attitudes and referral patterns of Lynch syndrome in Australia. Overall, our findings demonstrated suboptimal knowledge of Lynch syndrome. The presented fictitious individual—a female patient—has a remarkable family history of cancer representing that of revised Amsterdam II criteria. However, a third of clinicians, particularly GPs, did not consider the female patient to have high risk of colorectal or endometrial cancer (which is a concern as MMR mutation carriers have the highest lifetime risk for developing colorectal and/or endometrial cancer amongst the general population). In contrary, about half of all clinicians considered the female patient to have high risks of breast or ovarian cancers (which is another concern as MMR mutation carriers have up to 14% increased risk of ovarian cancer, and breast cancer has yet to be recognized as part of the Lynch syndrome spectrum of tumours) [4]. This suggests that clinicians could recognize the underlying genetic predisposition in the female patient, but lack familiarity with Lynch syndrome spectrum of tumours. However, this lack of familiarity with Lynch syndrome is consistent with other studies [29,38,39,40], reflecting the need to establish new ways to reach out to clinicians who are likely to diagnose, treat and survey Lynch families.

Parallel to this finding, we showed that GPs and gynaecologists were less familiar with IHC or MSI testing compared to the specialists. Nevertheless, this is expected, as only specialists apart from geneticist/genetic counsellor can initiate or order IHC or MSI tests directly from a pathology service. However, a proportion of specialists, including surgeons, was not familiar with IHC or MSI testing and was not certain if they were germline tests. The Royal College of Pathologists of Australia considers both IHC and MSI to be tests that assist in characterizing patient’s tumour, and do not constitute genetic testing for a familial disorder [41]. IHC and MSI testing are useful not only to identify patients with suspected Lynch syndrome but also to recognize suspected patients who do not meet the Amsterdam criteria for subsequent germline DNA sequencing. Both IHC and MSI provide predictive and prognostic information that may be used to guide treatment decisions. It is therefore important that clinicians know what these tests are so that accurate diagnosis and proper clinical management are warranted for at-risk patients and their family members to reduce cancer risk. Our above results raise concerns, as this implies that the clinicians might not know the purpose of IHC or MSI testing, and may not able to interpret the results correctly even when patients are tested. Thus, adoption of universal, or even age-selected, IHC testing of colorectal and endometrial tumours will reduce issues around poor identification of Lynch syndrome cases based on family-history selection criteria. However, there will still be need for targeted genetic education to improve physician understanding of IHC results, and subsequent referral of patients and their relatives for genetic testing and appropriate medical management.

Our results also show that clinicians were not familiar with cancer risk management associated with Lynch syndrome. Although the majority of clinicians recommended colonoscopy for high-risk patients, they also recommended gastroscopy, serum CA125, pelvic ultrasound and mastectomy. According to the eviQ Australian guideline (Appendix 2) and international recommendations [3,42], there is currently no evidence to support a survival benefit from gastroscopy, CA125 or pelvic ultrasound. Current recommendation for breast cancer, which is yet to be considered as part of Lynch syndrome tumour spectrum, is biannual mammography from age 45 or 50 years. Our results suggest that referral, if made, will not always be appropriate, as is unnecessary referral for screening and prophylactic surgeries.

While the majority of clinicians reported that they would assess family history and refer for genetic services when an asymptomatic individual present with a significant family history suggestive of Lynch syndrome, numerous barriers in collecting the information have been reported [43,44,45,46]. These include limitations of patients’ family history knowledge, time available for collection, knowledge and skills to collect and interpret family history data to provide appropriate risk assessment and clinical care recommendations. Nonetheless, it is important to collect a three-generation pedigree from individuals with a diagnosis of cancer, as this informs eligibility for mutation testing, and readily identifies members in the extended family who are eligible for predictive testing if a pathogenic mutation is identified in the proband. Published data have shown that time devoted on family history collection was often minimal in general practice [43], and inadequate even when documented for cancer patients [8,47,48,49].

Clinicians in our study considered patient disinterest to be a barrier to uptake of referral. Several published reports have demonstrated low rate of uptake of genetic counselling and testing by at-risk relatives (23% and 44%, respectively) [50,51]. An investigation by Wakefield *et al.* [52] involving 39 high-risk Australian patients for breast and/or ovarian cancer revealed positive attitude toward genetic counselling and testing but patient barriers to clinical attendance and testing uptake were poor understanding of cancer risk and eligibility for genetic testing [52]. It is possible that individuals at risk of Lynch syndrome may face similar challenges; however, no study to date has explicitly assessed Lynch cancer patients’ information needs. Our findings also suggest a slight preference by all respondents for the oncologists and GPs to make the initial referral for genetic services; however, a significant proportion of GPs did not feel that their role is to identify patients for genetics referral or to order IHC or MSI testing. As such, it is important to consider alternative methods to triage patients with suspected Lynch syndrome. Universal testing should be considered in Australia; however, to our knowledge such testing has yet to be formally established. Also, due to limited resources, more studies are needed to assess cost-effectiveness of universal testing prior to nationwide implementation.

In our previous study, we showed that a research-based self-administered family history questionnaire can be considered for use in specialist clinics to facilitate and improve family history documentation [8]. Indeed, a review has reported that a systematic family history tool may add significant family health information to current primary care practice [53], and a computer-based decision support system can further facilitate appropriate referral of high risk individuals for genetic services [54,55]. An automatic prompt for IHC or MSI testing may also be incorporated into such a system once routine universal testing is formally established in Australia to improve diagnosis and referral of patients for genetic services. Development of patient information brochure should further assist clinicians when consulting patients as well as to aid patients in making informed decisions about genetic testing.

There are several limitations to this study. Firstly, the response rate was low. This may be in part due to unwillingness to complete a web-based survey or lack of interest in hereditary cancer syndromes. A larger sample size and greater response rate would certainly provide a more accurate view on clinicians’ attitudes to genetics referral, however, the response rate in our study is comparable to other web-based studies conducted [56,57,58,59]. Selection bias may have occurred because participants had greater interest in hereditary cancer syndromes, more genomic knowledge, or stronger beliefs about their role in providing genetic services. Should this be true, our results would indicate even more strongly a need for improved education and support for clinicians to triage patients with suspected Lynch syndrome. Another limitation faced by our web-based data collection includes the inability to assess the representativeness of Australian healthcare providers due to lack of a national email registry, which may potentially bias our original sampling frame. We also did not know how many men and women within each practice category were asked for the survey initially, as the email broadcast was blinded by the Association for confidential reasons. Although clinicians were not evaluated for low or moderate risk Lynch syndrome-related clinical scenarios, the purpose of this study was to explore recognition of a classical family history suggestive of Lynch syndrome. Despite the abovementioned caveats, findings from this Australian study offer insight into clinicians’ knowledge of Lynch syndrome, and their attitudes toward genetic services and referral practices.

## 5. Conclusions

Our study suggested that genetics education is necessary for clinicians in order to improve genetic referral. Further studies should be conducted to examine educational topics selected by clinicians (e.g., genetic risk assessments, basic genomic concepts), and more behavioural research is needed to investigate barriers to patient uptake of referral as well as patients’ information needs. An online family history tool with built-in decision support for genetics referral would be helpful in triaging high-risk individuals in general practice. Future research should also focus on feasibility and impact assessment of reflex IHC or MSI and *MLH1* methylation testing to identify Lynch syndrome patients as a way to prevent second cancers, and identify carrier relatives for predictive testing.

## Appendix 1: Questionnaire

### Health Professionals’ Knowledge and Attitudes towards Genetic Services for Patients with or at High-Risk of Lynch syndrome

This survey is about health professionals’ knowledge and attitudes towards genetic services. Genetic services refer to the delivery of genetic counselling and risk assessment, and testing for patients and families with or at high-risk of hereditary syndrome.

### Demographic of Respondents

1. What is your gender?
  〇 Male
  〇 Female
2. What is your age?
  〇 18–29
  〇 30–39
  〇 40–49
  〇 50–59
  〇 60 +
3. What is your current specialty?
  〇 General practic
  〇 Gynaecology
  〇 Gastroenterology
  〇 Gynaecology oncology
  〇 Medical oncology
  〇 Radiation oncology
  〇 General surgery
  〇 Colorectal surgery
  〇 Other (please specify__________________)
4. How long have you been practicing in your current field?
  〇 0–5 year
  〇 6–10 year
  〇 11–20 year
  〇 More than 20 years
5. What is the postcode of your primary location of practice?
  Please specify _____________________

### You and Your Practice

The following section is about you and your referral practice. This is not a test. Your replies are anonymous and will be treated confidentially. Please answer all items.

6. In the past 12 months, have you referred patients for:
  	**Yes**	**No**	**Not sure**
  a. Genetic services?	❐^1^	❐^2^	
  b. DNA mutation testing?	❐^1^	❐^2^	❐^3^
  c. Tumour immunohistochemistry (IHC) testing	❐^1^	❐^2^	❐^3^
  d. Tumour microsatellite instability (MSI) testing	❐^1^	❐^2^	❐^3^
7. Do you consider the following testing a germline test?
  	**Yes**	**No**	**Not sure**
  a. IHC testing?	❐^1^	❐^2^	❐^3^
  b. MSI testing?	❐^1^	❐^2^	❐^3^

### Case Scenario

Please read the following patient background before answering Questions 8 and 9. This is not a test. Your replies are anonymous and will be treated confidentially. Please answer all items. If you are unfamiliar with a pedigree format and need more explanation, please read the paragraph next to the pedigree below.

8. In your medical opinion, what is Melissa’s risk for developing the following cancers as compared to the general population? (Please answer all items).
  	**Much higher**	**Somewhat higher**	**Same**	**Somewhat lower**	**Not sure**
  a. Breast cancer	❐^5^	❐^4^	❐^3^	❐^2^	❐^1^
  b. Gastric cancer	❐^5^	❐^4^	❐^3^	❐^2^	❐^1^
  c. Ovarian cancer	❐^5^	❐^4^	❐^3^	❐^2^	❐^1^
  d. Thyroid cancer	❐^5^	❐^4^	❐^3^	❐^2^	❐^1^
  e. Colorectal cancer	❐^5^	❐^4^	❐^3^	❐^2^	❐^1^
  f. Endometrial cancer	❐^5^	❐^4^	❐^3^	❐^2^	❐^1^
9. About a year later, Melissa was diagnosed with endometrial cancer. How would you proceed?
  	**Very likely**	**Somewhat likely**	**Not likely**	**Very unlikely**	**Not sure**
  a. Offer surveillance	❐^5^	❐^4^	❐^3^	❐^2^	❐^1^
  b. Referral to a geneticist	❐^5^	❐^4^	❐^3^	❐^2^	❐^1^
  c. Family history assessment	❐^5^	❐^4^	❐^3^	❐^2^	❐^1^
  d. Discussion of risk-reducing surgery	❐^5^	❐^4^	❐^3^	❐^2^	❐^1^
  e. Referral to a non-genetics specialist	❐^5^	❐^4^	❐^3^	❐^2^	❐^1^
  f. Order genetic testing for germline mutations	❐^5^	❐^4^	❐^3^	❐^2^	❐^1^
  g. Order pre-genetic testing of tumour tissue (e.g., MSI or IHC)	❐^5^	❐^4^	❐^3^	❐^2^	❐^1^
  h. Discussion about Lynch syndrome cancers with patient	❐^5^	❐^4^	❐^3^	❐^2^	❐^1^
  i. No action	❐^5^	❐^4^	❐^3^	❐^2^	❐^1^
10 If you have chosen to offer surveillance, what would you offer? (check all that apply)
  ❐ Colonoscopy
  ❐ Gastroscopy
  ❐ CA125
  ❐ Mammography
  ❐ Other, please specify _____________________
11. If you have chosen to discuss risk-reducing surgery with patients, what kind of risk-reducing surgery would you discuss with the patient?
  Please specify _____________________

### General Attitudes about Genetic Services

The flowing section is about why you would or would not refer patients for genetic services. We would like you to answer the questions whether or not you currently use genetic services.

12. In your opinion, which of the following factors play a role in your decision to refer a patient for genetic services? (Check all that apply)
  ❐1 Patient interest or request
  ❐2 Ethical and legal responsibility
  ❐3 Reassurance for the patient and family
  ❐4 To provide genetic counselling for the patient
  ❐5 Genetic testing for germline mutations
  ❐6 To provide appropriate cancer risk assessment for the patient
  ❐7 To provide appropriate screening and management for the patient
  ❐8 To provide appropriate screening and/or management for the patients’ family
  ❐9 Other, please specify: ________________________________
13. In your opinion, which of the following factors play a role in your decision to NOT refer a patient for genetic services? (Check all that apply)
  ❐1 I do not feel it is my responsibility
  ❐2 I do not have access to a genetic health service
  ❐3 Patients may be at risk for insurance discrimination
  ❐4 Long waiting time for appointment at a genetic clinic
  ❐5 Patient was not interested when referral was offered
  ❐6 I do not feel familiar with hereditary cancer syndromes
  ❐7 Patient is unlikely to benefit from genetic counselling and/or testing
  ❐8 I do not know how to make a referral to the local genetic health service
  ❐9 Recommendations and guidelines are not available to select patients for referral
  ❐10 Other, please specify: ________________________________________
14. Who do you think is best to refer patients for genetic services (*i.e*., to make the initial offer)? (Please choose only one of the following, and provide the reason(s) for your selection)
  〇 General practitioners
  〇 Gynaecologists
  〇 Gastroenterologists
  〇 Gynaecology oncologists
  〇 Medical oncologists
  〇 Radiation oncologists
  〇 Colorectal surgeons
  〇 General surgeons
  〇 Any of the above
  〇 Make a comment on your choice here: ______________________________
15. When is the best time for the initial referral for genetic services to be made to the patient? (Please choose only one of the following, and provide the reason(s) for your selection)
  〇 When family history is collected
  〇 At diagnosis of cancer
  〇 After surgery and before commencement of adjuvant therapy
  〇 During adjuvant therapy
  〇 After treatment is finished
  〇 At any time
  Make a comment on your choice here: ______________________________
16. I feel my role as a physician includes: (Check all that apply)
  ❐1 Providing emotional support after genetic testing
  ❐2 Identifying patients for referral to genetic services
  ❐3 Interpreting germline DNA-based genetic test results
  ❐4 Collecting a three-generation family history information
  ❐5 Ordering pre-genetic testing of tumour tissue (e.g. MSI or IHC)
  ❐6 Counselling patients about their cancer risks **after** genetic testing
  ❐7 Counselling patients about their cancer risks **before** genetic testing
  ❐8 Calculating relative risk of cancer associated with family cancer history
  ❐9 Discussing the need for cancer surveillance or prophylaxis with patients when required
  ❐10 Providing regular clinical examination and care to patients with hereditary cancer syndromes
  ❐11 Other, please specify: ______________________________
17. In your opinion, which of the following would support your practice? (Please choose the appropriate response for each item)
  	**Very likely**	**Somewhat likely**	**Not likely**	**Very unlikely**	**Not sure**
  a. Clear referral guideline	❐^5^	❐^4^	❐^3^	❐^2^	❐^1^
  b. Online risk assessment tool	❐^5^	❐^4^	❐^3^	❐^2^	❐^1^
  c. Continuing genetic education programmes	❐^5^	❐^4^	❐^3^	❐^2^	❐^1^
  d. Genetic information brochure for patients	❐^5^	❐^4^	❐^3^	❐^2^	❐^1^
  e. A coordinator in clinic to collect patient family history	❐^5^	❐^4^	❐^3^	❐^2^	❐^1^
  f. A genetic counsellor in clinic to assess risk and to facilitate referral	❐^5^	❐^4^	❐^3^	❐^2^	❐^1^
  g. A computerized family history tool with decision support for referral	❐^5^	❐^4^	❐^3^	❐^2^	❐^1^
  h. The presence of a genetic counsellor in a multidisciplinary tumour board meeting	❐^5^	❐^4^	❐^3^	❐^2^	❐^1^
18. **Is there anything else you would like to tell us about**?
19. **If you would like to receive a copy of the research summary report, please enter your email address here**: _______________________________________________
